# A systematic review of auricular therapy for poststroke cognitive impairment and dementia: A protocol for systematic review and meta-analysis

**DOI:** 10.1097/MD.0000000000032933

**Published:** 2023-02-17

**Authors:** Zhaohong Gao, Junfeng Li, Liqin Wang, Yan Li

**Affiliations:** a First Affiliated Hospital, Heilongjiang University of Chinese Medicine, Harbin, China.

**Keywords:** auricular therapy, meta-analysis, protocol, PSCID, systematic review

## Abstract

Post-stroke cognitive impairment and dementia (PSCID) is the main source of morbidity and mortality after stroke worldwide. It is one consequence of ischemic stroke, intracerebral hemorrhage, or subarachnoid hemorrhage. Cognitive impairment and dementia after the clinical stroke may contribute to the clinical expression of PSCID, which are prevalent clinical symptoms, especially in the elderly. Current problems in the field of PSCID are related to a lack of harmonization of the classification and definition, deficiency of well-defined diagnosis, deficiency of standardized and objective treatment plans, etc. Auricular therapy can effectively improve the symptoms of patients with PSCID. However, there has been no systematic review of auricular therapy for PSCID. This study aimed to evaluate the efficacy and safety of auricular therapy in patients with PSCID.

**Methods:**

Before December 2022, a systematic literature search was conducted using the following databases: PubMed, Embase, SinoMed (previously called the Chinese Biomedical Database), Web of Science, Chinese National Knowledge Infrastructure, and Wanfang Database. Review Manager software (version 5.3) will be used for statistical analysis; otherwise, descriptive analysis or subgroup analysis will be conducted. The quality of evidence for outcomes will be assessed with the Grading of Recommendations Assessment, Development and Evaluation approach.

**Results:**

This meta-analysis further confirmed the beneficial effects of auricular therapy in patients with PSCID.

**Conclusion:**

This study investigated the efficacy and safety of auricular therapy in patients with PSCID, providing clinicians and patients with additional options for this disease.

## 1. Introduction

Stroke is one main cause of disability.^[1]^ Cognitive impairment and dementia as important aspects for stroke survivors used to be neglected while interventions and researchers pretended to focus on physical disabilities.^[2]^ Current evidence suggests that poststroke cognitive impairment and dementia (PSCID) may affect up to one-third of stroke survivors.^[3,4]^ According to the existing clinical data, there are 2 different stages of PSCID, including poststroke cognitive impairment (PSCI) and poststroke dementia (PSD), which has a significantly higher mortality rate than the previous one.^[5]^

Some researchers use the terms early and late (or delayed) PSCID to differentiate the cognitive deficits detected in the immediate poststroke period from those that develop over the proceeding months.^[6]^ That is not a problem when the researchers try to explain the nature of the relevant defects in the clinical study. However, there is a lack of consensus in terms of disease definition. Therefore, these distinctions are arbitrary and inaccurately defined. Generally, PSCID encompasses cognitive impairments manifesting in the 3 to 6 months after incident stroke, which are similar to canonical vascular cognitive impairment (VCI) or vascular dementia (VD), and are frequently detected on poststroke cognitive screening. Hence, PSCID is often conflated with VCI and VD. Since the diagnosis of PSCID is not completely clear, in this article, PSCI has been defined as all problems in cognitive function that occur following a stroke, irrespective of the (stroke) etiology according to the European Stroke Organisation and European Academy of Neurology.^[7]^ By contrast, poststroke dementia is defined as immediate and/or delayed cognitive decline that begins within 6 months after a stroke and that does not reverse (encompasses dementia that develops within 6 months of stroke in patients).^[8]^

PSCID has great clinical heterogeneity. The cause of the disease is stroke, which may also be affected by neurodegenerative diseases. Therefore, the treatment and management of PSCID mainly refer to vascular cognitive impairment and neurodegenerative diseases.^[9]^ In addition to actively treating acute stroke, effective secondary prevention, such as medical intervention, and lifestyle change, and nonpharmacological therapies such as auricular therapy also effective in the prevention and treatment of some symptoms of PSCID.^[10,11]^

Auricular therapy has over 2000 years of history of use in China, and Paul Nogier presented the inverted fetus map to describe the holographic theory in 1957, which makes it possible to understand the theory of auricular therapy systematically and comprehensively.^[12]^ Since then, auricular therapy has become one of the most popular therapeutic methods in many Western countries.^[13]^ The manipulation of auricular therapy is based on the holographic theory, a sort of assumption that information regarding a part of the entire organism could be retrieved from the corresponding point of the ear so that stimulation to a specific point of the ear could ameliorate the function of the corresponding visceral organ or another part of the body.^[14]^ Nogier believed that the underlying mechanism behind the connections between a part of the body and a point of the ear is related to the autonomic nervous system.^[12]^

Auricular therapy is extensively applied by both doctors and nurses worldwide as a preventive-therapeutic method for certain internal diseases including PSCID.^[15,16]^ Although many clinical studies have been conducted to investigate the effectiveness of auricular therapy for treating or controlling PSCID, these studies have not yet been systematically summarized and the overall evidence is still uncertain. Herein, it is worthwhile to evaluate the evidence for the effectiveness and safety of auricular therapy for PSCID with a systematic review and to provide recommendations on future research and practice in this field.

## 2. Methods

The system evaluation registration number (CRD42023387072) was registered in the PROSPERO International System Evaluation Prospective Registry. Which will be performed following the guideline of Preferred Reporting Items for Systematic Review and Meta-Analysis Protocols (PRISMA-P).^[17]^ All steps of this systematic review will be performed according to the Cochrane Handbook (5.3.0).^[18]^

### 2.1. Eligibility criteria

#### 2.1.1. Type of study.

We will include all randomized controlled trials (RCTs) regarding auricular therapy for PSCID. Nonrandomized clinical studies, cluster randomized trials, and animal experiments will be excluded. The language is limited to English and Chinese.

#### 2.1.2. Types of participants.

Participants had been diagnosed with PSCID, regardless of sex, age, race, education, disease course, and severity, and met the following criteria:

a. Diagnostic criteria of PSCI: all problems in cognitive function that occur following a stroke, irrespective of the (stroke) etiology.
b. Diagnostic criteria of PSD: immediate and/or delayed cognitive decline that begins within 6 months after a stroke and that does not reverse (encompasses dementia that develops within 6 months of stroke in patients).

#### 2.1.3. Types of interventions and comparisons.

The main intervention method in this study was auricular therapy, which also included auricular needling, ear massage, auricular pressure seeds, and other similar methods. Trials that combined auricular therapy and other treatments were also included. There was no limit to the time or frequency of auricular therapy. Patients in the control group received acupuncture, drugs, placebos, or no treatment.

#### 2.1.4. Types of outcome measures.

The outcome measures include the recovery of cognitive function, improvement of mental symptoms, and improvement of daily living ability, etc. The primary outcome measures include Mini-Mental State Examination (MMSE), Montreal Cognitive Assessment (MoCA), Loewenstein (LOTCA), and Modified Barthel Index (MBI). Secondary outcome measures include Neurobehavioral Cognitive Status Examination (NCSE), auditory verbal learning test (AVLT), Stroop color and word test (SCWT), trail-making test (TMT), Boston naming test (BNT), digital span test (DST), clinical dementia rating scale (CDR), Wechsler memory scales (WMS), etc.

### 2.2. Exclusion criteria

The exclusion criteria contain the following items:

1. a. The above diagnostic criteria are not met.
2. b. Dementia due to Alzheimer disease or other causes.
3. c. Suffering from diseases that can cause dementia as a complication and other illnesses that can interfere with the continuous and effective conduct of the experiment.
4. d. Other diseases that affect cognitive function.

### 2.3. Search strategy

Our research group plans to search PubMed, Embase, SinoMed (previously called the Chinese Biomedical Database), Web of Science, Chinese National Knowledge Infrastructure, and Wanfang Database. From the beginning of the study to December 2022, all published RCTs in Chinese and English were included. The retrieval mode used will be a combination of free words and medical subject headings terms, including “poststroke cognitive impairment and dementia,” “poststroke cognitive impairment,” “poststroke dementia,” “cognitive impairment after stroke,” “dementia after stroke,” “auricular therapy,” “auricular needling,” “auricular massage,” “ear pressure seeds.” The search strategy used PubMed as an example (Table 1). In addition, the reference lists of previously published systematic reviews were manually examined for further pertinent studies.

**Table 1:** Search strategy.

Number	Terms
#1	Auricular therapy
#2	Auricular needling
#3	Auricular acupuncture
#4	Auricular plaster
#5	Auricular pressure seeds
#6	Therapy, Auricular
#7	Ear points
#8	Ear needling
#9	Ear acupuncture
#10	Ear massage
#11	Ear pressure seeds
#12	Otopoint
#13	#1 OR #2-#12
#14	Post-stroke cognitive impairment and dementia
#15	Post-stroke cognitive impairment
#16	Post-stroke dementia
#17	Cognitive impairment after stroke
#18	Dementia after stroke
#19	#14 OR #15-#18
#20	Randomized controlled trial
#21	Controlled clinical trial
#22	Randomized
#23	Randomly
#24	Random allocation
#25	Placebo
#26	Double-blind method
#27	Single-blind method
#28	Trials
#29	Clinical trials
#30	#20 OR #21-#29
#31	#13 And #19 And #30

### 2.4. Data collection and analysis

#### 2.4.1. Selection of studies.

**Figure 1.**
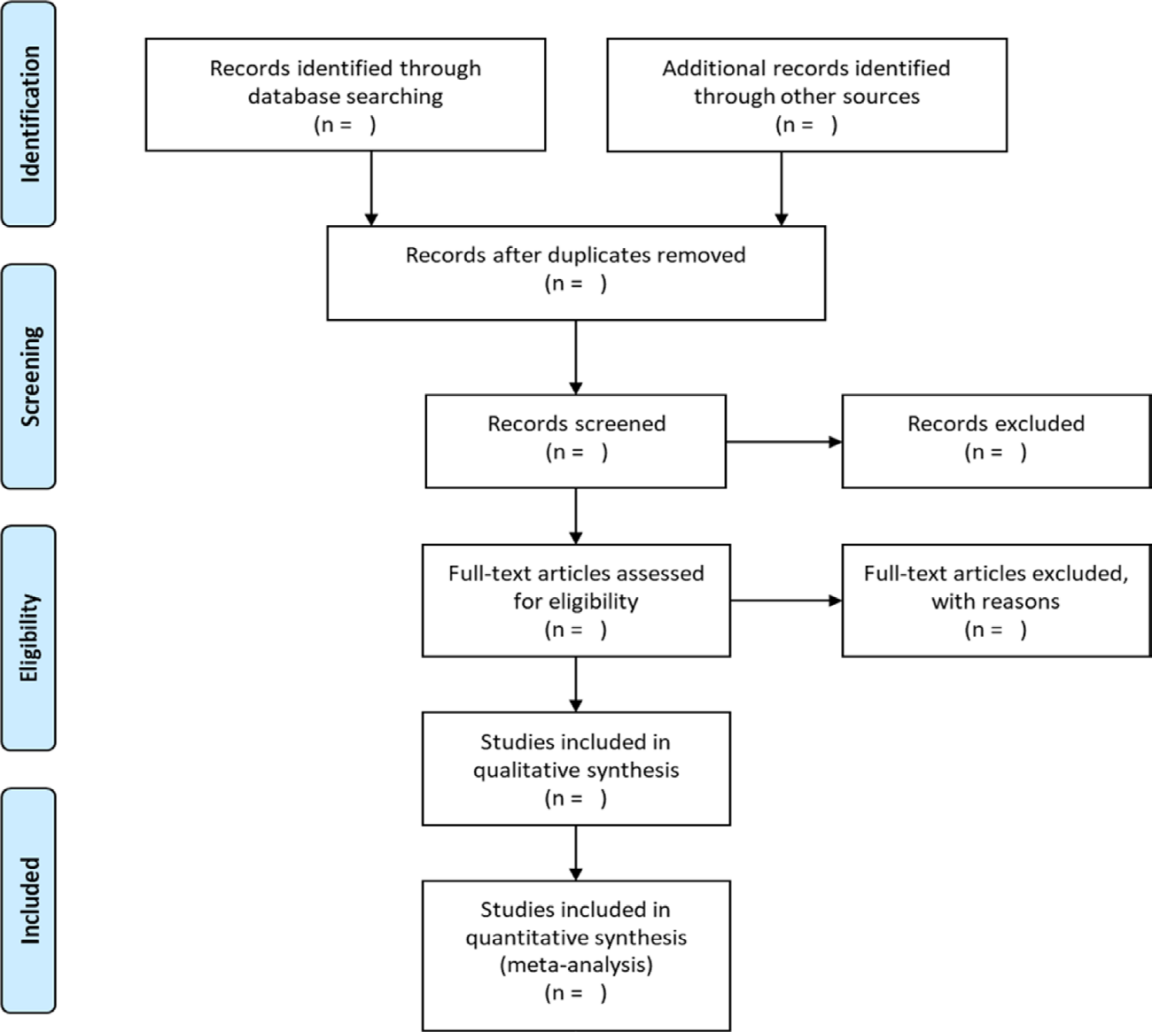
Flow chart of study selection.

First, 2 members (ZG, JL) of the research group will independently screen eligible studies. The selected research was imported into the document management software EndNote X9. Second, preliminary screening was conducted according to the title and abstract, and the appropriate research was selected according to the inclusion criteria. Finally, it was decided that if there were differences in inclusion and exclusion, the conclusion would be drawn through group discussions. This process is shown in the PRISMA follow-up diagram (Fig. 1).

#### 2.4.2. Data extraction and management.

The literature data extraction will be done independently by 2 researchers (ZG and JL) and all data will be extracted and tabulated using a standardized data extraction form. The extracted data included the author`s name, title, country, publication date, research design, age and sex of participants, course of the disease, details of control interventions, time, results, follow-up, adverse effects, and any other relevant information. The consistency of the data will be checked by a third researcher (LW), and if the required information is incomplete, it will be obtained by contacting the original author whenever possible.

#### 2.4.3. Dealing with missing data.

In the face of missing data or unclear research, we contacted the relevant authors to obtain missing information before making an exclusion decision. If true information acquisition failed, we excluded it from the analysis.

### 2.5. Risk of bias assessment

The Cochrane^[19]^ Collaboration tool was used to assess the risk of bias in each study, including the following 6 types of bias: random sequence generation, allocation concealment, participant and personal blinding, outcome assessment blinding, incomplete outcome data, selective reporting, and other sources of bias. The quality of the report was divided into 3 levels: low, unclear, and high-risk. Differences were resolved through group discussion.

### 2.6. Statistical analysis

This study will be analyzed using RevMan version 5.3 (The Nordic Cochrane Centre for The Cochrane Collaboration,Copenhagen, Denmark). Relative risk (RR) was used when the results were dichotomous variables with 95% confidence intervals. For continuous variables, we used the standardized mean difference and 95% confidence intervals. The chi-square test and *I*^2^ statistic will be used to confirm heterogeneity. The former checks for heterogeneity, whereas the latter reflects the degree of heterogeneity through a specific value. *I*^2^ values of 25%, 50%, and 75% indicated low, medium, and high heterogeneity, respectively. When *I*^2^ > 50%, *P* < .10, it indicates that there is more heterogeneity among the studies and the source of heterogeneity will be analyzed and the random-effect model will be applied. For the research results with large heterogeneity that cannot be quantitatively integrated, a narrative report will be made.

### 2.7. Subgroup analysis

If heterogeneity was obvious, according to the characteristics of this study, subgroup analysis was performed to explore the sources of heterogeneity from the aspects of age, sex, region, control intervention type, and type of auricular therapy.

### 2.8. Sensitivity analysis

In terms of quality analysis, we conducted a sensitivity analysis on the main results to explore the impact of individual research bias on the results.

### 2.9. Grading the quality of evidence

We’ll use the Grading of Recommendations Assessment, Development, and Evaluation method for evaluation. The evaluation included bias risk; heterogeneity; indirectness; imprecision; publication bias. The quality of evidence for included RCTs will be divided into high, medium, low, and very low.

### 2.10. Ethics and dissemination

Since this is a protocol of systematic review and meta-analysis, ethics approval is not required. We will report our findings of this systematic review and meta-analysis in a peer-reviewed journal in the future.

## 3. Discussion

As we mentioned earlier, PSCID is similar to canonical vascular cognitive impairment and dementia. While abundant and various progress has been made in the processing of conceptualizing vascular cognitive impairment and dementia,^[20]^ the accurate mechanisms of PSCID are insufficiently understood.^[21]^ Besides, there is no pharmacological treatment approved for PSCID currently. Auricular therapy, as a traditional external therapy in traditional Chinese medicine, is one safe operation and high acceptance of treatment by patients. There have been some clinical observations that have confirmed that this therapy has positive effects on the cognitive function, daily living ability, and intelligence status of PSCID patients, and even related brain functions such as promoting the recovery of vascular endothelial function.^[22,23]^ Our research team will objectively and comprehensively evaluate the therapeutic effect of auricular therapy on PSCID to improve the recovery of cognitive function, improvement of mental symptoms, and improvement of daily living ability. The results of this review will provide neurologists, psychologists, and patients with more information on treatment options for PSCID and new directions for future research based on the credibility of existing evidence.

## Author contributions

**Conceptualization:** Zhaohong Gao, Junfeng Li.

**Data curation:** Zhaohong Gao, Junfeng Li, Yan Li.

**Formal analysis:** Zhaohong Gao.

**Funding acquisition:** Liqin Wang.

**Investigation:** Zhaohong Gao, Junfeng Li.

**Methodology:** Yan Li.

**Project administration:** Liqin Wang.

**Writing – original draft:** Zhaohong Gao.

**Writing – review & editing:** Zhaohong Gao, Junfeng Li, Liqin Wang.
